# Electrophysiological findings in long-term type 1 diabetes patients without diabetic retinopathy using different ERG recording systems

**DOI:** 10.1038/s41598-024-54099-5

**Published:** 2024-02-12

**Authors:** Marta Arias-Alvarez, Cristina Tomas-Grasa, Maria Sopeña-Pinilla, Elvira Orduna-Hospital, Guisela Fernandez-Espinosa, Sofia Bielsa-Alonso, Javier Acha-Perez, Diego Rodriguez-Mena, Isabel Pinilla

**Affiliations:** 1 Department of Neurophysiology, Lozano Blesa University Hospital, 50009 Zaragoza, Spain; 2 Aragon Institute for Health Research (IIS Aragon), 50009 Zaragoza, Spain; 3 Department of Internal Medicine, Lozano Blesa University Hospital, 50009 Zaragoza, Spain; 4 Department of Ophthalmology, Miguel Servet University Hospital, 50009 Zaragoza, Spain; 5 Department of Applied Physics, University of Zaragoza, 50009 Zaragoza, Spain; 6 Department of Endocrinology, Miguel Servet University Hospital, 50009 Zaragoza, Spain; 7 Department of Surgery, University of Zaragoza, 50009 Zaragoza, Spain; 8 Department of Ophthalmology, Lozano Blesa University Hospital, 50009 Zaragoza, Spain

**Keywords:** Neuronal physiology, Predictive markers

## Abstract

To assess full-field electroretinogram findings in long-term type 1 diabetes patients without diabetic retinopathy. Prospective study including 46 eyes of 23 patients with type 1 diabetes and 46 age-matched healthy eyes evaluated by the RETI-port/scan21 and the portable system RETeval following ISCEV guidelines. The average duration of diabetes was 28.88 ± 8.04 years. In scotopic conditions, using the RETI-port/scan21, diabetic patients showed an increase in b-wave implicit time (IT) (p = 0.017) with the lowest stimuli; a diminished b-wave amplitude (p = 0.005) in the mixed response, an increased IT (p = 0.004) with the high-intensity stimuli and an OP2 increased IT (p = 0.008) and decreased amplitude (p = 0.002). Under photopic conditions, b-wave amplitude was lower (p < 0.001) and 30-Hz flicker response was diminished (p = 0.021). Using the RETeval, in scotopic conditions, diabetic patients showed a reduction in the rod b-wave amplitude (p = 0.009), an increase in a-wave IT with the 280 Td.s stimulus (p = 0.005). OP2 had an increased IT and diminished amplitude (p = 0.003 and p = 0.002 respectively). 16 Td.s flicker showed an increased IT (p = 0.008) and diminished amplitude (p = 0.048). Despite variations in values between both systems, nearly all results displayed positive correlations. Long-term type 1 diabetes patients without diabetic retinopathy exhibit alterations in scotopic conditions, as evidenced by both conventional and portable electroretinogram devices. These findings suggest a modified retinal function, particularly in rod-driven pathways, even in the absence of vascular signs.

## Introduction

Diabetic retinopathy (DR) is the leading cause of visual loss in the working-age population. Its prevalence has persistently increased over the years, representing a significant cost for health care systems worldwide^1,2^. DR is a complex disease that affects not only the retinal microvasculature but also neurons and glial cells^3^. Neurodegeneration is known to have an important role in the development of the disease, and DR is considered to be a neurovascular complication of diabetes mellitus^4^. Early dysfunction of the neurovascular units has been described in DR, which can lead to impaired function, such as in the response to functional hyperaemia^5^. The main structural finding prior to the onset of DR is the diminution of the ganglion cell complex and retinal nerve fibre layer thickness^6,7^. Apoptosis of both ganglion and amacrine cells has been described as the first neural change induced by diabetes mellitus, as has reactive gliosis^8,9^. Functional tests have shown abnormalities before the onset of DR, including colour perception^10^, contrast sensitivity^11^, dark adaptation^12^, visual fields^13^, macular sensitivity^14,15^ and neurophysiological tests^16–18^.

Full-field electroretinography (ffERG) is designed to record the massive response of the retina to light stimulation. The role of neurodegeneration adds new value to electrophysiology in the diagnosis of DR. Changes in ERG have been described in patients with diabetes mellitus, becoming more pronounced as the severity of the DR increases^16,18–20^. In recent years, different devices have been used to screen for DR. RETeval (LKC Tech. Inc., Gaithersburg, MD, USA) is a portable system that measures pupillary responses and performs ffERG without mydriasis. It has been shown that it is a valid method for screening vision-threatening DR^21,22^. To the best of our knowledge, RETeval results in diabetes mellitus patients have not been compared to their standard ERG results.

The aim of our study was to analyse the ERG response in long-term type 1 diabetes patients without DR and in an age-matched control group using RETeval and to compare the results with those of a conventional ERG system.

## Methods

This study was conducted in accordance with the principles outlined in the Declaration of Helsinki and received approval from the Ethics Committee for Clinical Research of Aragon (CEICA PI22/587). Prior to any examinations, written informed consent was obtained from each participant.

A prospective observational study on visual function in 46 eyes from 23 type 1 diabetes patients and 46 eyes from 23 normal age-matched subjects was carried out from October 2022 to May 2023 using ffERG. All patients were evaluated at the Neurophysiology Department of the Lozano Blesa University Hospital by the same investigator.

The type 1 diabetic patients were monitored by the Endocrinology Unit. HbA1c, lipid values and arterial blood pressure remained well controlled. All participants underwent an ophthalmological and neurophysiological examination that included medical history, best-corrected visual acuity (BCVA), axial length (AL) using IOLmaster 500 (Carl Zeiss Company, Jena, Germany), slit-lamp examination, intraocular pressure (IOP) measurement, fundus examination and wide-field retinography with the Clarus 500 (Carl Zeiss Meditec, Dublin, USA), macular thickness using Spectralis optical coherence tomography (OCT) (Heidelberg Engineering, Heidelberg, Germany), and ffERG.

The inclusion criteria for the diabetic group were a diagnosis of type 1 diabetes at least 15 years prior, no clinical signs of DR, BCVA better than 20/25 on the Snellen chart with refractive errors less than 5D of spherical equivalent or 3D of astigmatism and signing the informed consent form. The control group was made up of healthy subjects similar in age to the diabetic group, who met and the same inclusion criteria except for the diagnosis of diabetes mellitus.

The exclusion criteria for both the diabetic and control groups were the presence of any sign of DR or other ophthalmologic pathology, IOP over 21 mmHg on Goldmann tonometry, optic nerve pathology, ocular inflammation, and any previous ocular surgery.

For the ERG tests, two recording systems were employed: the RETI-port/scan21 (Roland Consult, Brandenburg, Germany) and the handheld ERG RETeval. First, the ERG examination was conducted using the RETI-port/scan21 system (Version 1021.3.0.0). Following the protocols established by the International Society for Clinical Electrophysiology of Vision (ISCEV)^23^, scotopic and photopic ffERG was performed. The ISCEV band-pass (0.3–300 Hz) without notching filtering was employed for both systems.

To ensure consistent pupil dilation, 1% tropicamide eye drops were instilled. Simultaneous recordings were made for both eyes.

After topical anaesthesia, active sterile DTL electrodes were carefully positioned across the bulbar conjunctiva. A gold-cup skin reference electrode was placed superotemporally or laterally to the orbital rim, while a ground electrode was positioned on the forehead.

Subjects were instructed to focus on the central fixation red LED light, while the examiner monitored fixation on the video monitor.

Following a minimum dark adaptation period of 20 min, four scotopic responses were recorded: dark-adapted (DA) response to 0.01 cd.s/m^2^ or rod response, DA response to 3.0 cd.s/m^2^ or mixed cone-rod response, DA response to 10.0 cd.s/m^2^ or mixed cone-rod response with high-intensity flash; additionally, another oscillatory potential (OP) response to 3.0 cd.s/m^2^ (second-order OP) was obtained as a separate recording. Subsequently, two photopic responses were recorded after a minimum light adaptation period of 10 min: light-adapted (LA) response to 3.0 cd.s/m^2^ or cone response and LA response to 3.0 cd.s/m^2^ at 30 Hz or selective cone response.

The amplitudes (μV) and implicit times (IT) (ms) of each recorded waveform were measured and analysed.

After 2 days, ERG examination was conducted using the portable RETeval ERG system. A special skin electrode array (Sensor Strip, LKC Technologies, Inc.) was positioned on the orbital rim, 2 mm from the margin of the lower eyelid. This array comprises three electrodes: an active electrode (positive), a reference electrode (negative), and the ground electrode, all within a single adhesive tape. While skin electrodes have traditionally been avoided in ERG testing due to lower signal levels^24^, advancements in hardware data acquisition and Fourier-based analysis methods have facilitated the reproducible results outlined in this study, eliminating the need to use corneal electrodes. Two protocols were employed: the 6-step ISCEV protocol, followed by the DR Assessment protocol.

Participants underwent ERG without pupil dilation. The RETeval device adjusts the flash luminance to deliver the desired amount of light into the eye, regardless of the pupil size. We assumed the pupil diameter was 6 mm to convert standard ISCEV dilated luminances to Trolands.

The responses of both eyes were measured monocularly. Subjects were instructed to focus on the fixation spot and to open their eyelids wider to enhance pupil visibility. The recording began with the LA responses: the LA response to 85 Td.s (cone response) and the LA response to 85 Td.s at 28.3 Hz or selective cone response. After 20 min of dark adaptation, the DA steps of the ISCEV protocol were performed: the DA response to 0.28 Td.s or rod response, the DA response to 85 Td.s (mixed cone-rod response, the DA response to 280 Td.s or mixed cone-rod response with high intensity, and a separate DA response to 85 Td.s to evaluate the OP. We added 2 more steps from the DR Assessment protocol: the stimulus flash illuminance at 16 and 32 Td.s at 28.3 Hz with no background. The protocol combined IT, amplitude, age, and pupil response to generate a DR risk assessment score for each eye. The default normal values ranged from 7 to 19.9. Cut-off values of 20.75 and 23.05 were set for defining DR and vision-threatening DR, respectively^25,26^.

The amplitudes (μV) and implicit times (ms) of each recorded waveform were measured. In the OP, 5 OPs were evaluated.

### Statistics

All data were collected in an Excel spreadsheet (Microsoft Office Excel 2011, Microsoft Corporation, Redmond, WA, USA). Statistical analysis was performed using the Statistical Package for the Social Science (SPSS) 22.0 (SPSS Inc., IBM Corporation, Sommers, NY, USA). Normal distribution was studied using the Kolmogorov‒Smirnov test. After checking that the data did not follow a normal distribution, nonparametric tests were performed (Mann‒Whitney *U* test) for independent samples to assess differences between groups. To compare results between the parameters of both ERG devices, the Wilcoxon signed-rank test was used. Spearman's rho was used to calculate the bivariate correlation coefficients. The Bonferroni correction was applied for multiple comparisons at each intensity stimulus, and the p-value was determined to assess statistical significance. To evaluate inter-eye correlation, we used the intraclass correlation coefficient (ICC)^27,28^. Correlation was assessed in the amplitude of the a and b wave for the mixed scotopic response in both DM and control eyes.

## Results

Mean age was 48 ± 9.77 years (range 28–69) and 51.7 ± 4.75 years (range 40–59) in the type 1 diabetes patients and in the control group, respectively, with no differences between groups. Both the type 1 diabetes and control groups were 14 females (60.8%) and 9 males (39.13%). The average duration of the disease was 28.88 ± 8.04 years (range 18–47). Age at diagnosis was 17.96 ± 13.43 (range 2–47). The diabetic group had a mean HbA1c value of 7.29 ± 0.89% (range 6–8). Blood pressure and lipid values all stayed within normal limits (Table 1). BCVA was lower in the diabetic group (0.03 ± 0.06 LogMAR) than the control group (− 0.01 ± 0.04 LogMAR) (p = 0.001). IOP was similar in both groups (15.76 ± 2.14 and 15.75 ± 3.42 in the diabetic and control groups, respectively) (p = 0.614).

**Table 1:** Mean and standard deviation (SD) of the metabolic characteristics of the diabetic group related to the duration and control of the disease.

Type 1 diabetes group	Mean ± SD
Duration of diabetes (years)	28.88 ± 8.04
Age at diagnosis (years)	17.96 ± 13.43
HbA1c (%)	7.29 ± 0.89
Glycaemia (mg/dL)	149.00 ± 66.13
Total cholesterol (mg/dL)	190.61 ± 33.15
HDL cholesterol (mg/dL)	62.43 ± 12.38
LDL cholesterol (mg/dL)	114.30 ± 27.78
Urea (mg/dL)	34.35 ± 8.62
Creatinine (mg/dL)	0.78 ± 0.10
Albumin/creatinine ratio (mg/g Cr)	7.13 ± 10.19

*HbA1c* glycosylated haemoglobin, *HDL* high-density lipoprotein, *LDL* low-density lipoprotein, *SD* standard deviation.

### RETI-port/scan21 results: (Table 2)

**Table 2:** ERG values obtained with the Roland ERG following the ISCEV protocol in the diabetic and control groups.

		Control group n = 46	DM1 group n = 46	p Mann–Whitney *U* test	Bonferroni correction
		Mean ± SD	Mean ± SD		
Scotopic ERG					
DA 0.01	b-wave (ms)	87.31 ± 5.54	91.81 ± 9.88	0.017	**0.017**
	b-wave (µV)	230.29 ± 85.25	194.88 ± 77.46	0.036	NS
DA 3.0	a-wave (ms)	19.92 ± 2.83	19.71 ± 3.09	0.602	NS
	a-wave (μV)	− 207.09 ± 64.06	− 183.61 ± 57.06	0.083	NS
	b-wave (ms)	47.35 ± 4.05	48.03 ± 4.41	0.382	NS
	b-wave (µV)	387.53 ± 124.48	314.38 ± 94.28	0.005	**0.005**
	b/a	1.85 ± 0.28	1.76 ± 0.31	0.231	NS
DA 10.0	a-wave (ms)	15.89 ± 0.75	16.47 ± 1.59	0.023	NS
	a-wave (μV)	− 249.18 ± 78.29	− 231.07 ± 71.54	0.247	NS
	b-wave (ms)	45.39 ± 4.21	48.29 ± 6.64	0.004	**0.004**
	b-wave (μV)	377.75 ± 131.06	331.20 ± 103.97	0.091	NS
	b/a	1.53 ± 0.19	1.49 ± 0.20	0.674	NS
OP	OP2 (ms)	24.38 ± 0.68	24.87 ± 0.83	0.008	**0.008**
	OP2 (μV)	64.23 ± 18.49	52.28 ± 15.96	0.002	**0.002**
Photopic ERG					
LA 3.0	a-wave (ms)	14.96 ± 1.17	14.71 ± 1.40	0.209	NS
	a-wave (μV)	− 41.52 ± 12.85	− 35.16 ± 11.72	0.036	NS
	b-wave (ms)	32.15 ± 1.34	31.69 ± 1.24	0.146	NS
	b-wave (µV)	166.06 ± 51.00	123.62 ± 46.78	< 0.001	** < 0.001**
30 Hz flicker	N1P1 (ms)	29.50 ± 1.86	28.83 ± 1.02	0.160	NS
	N1P1 (μV)	123.53 ± 35.90	104.30 ± 29.14	0.021	**0.021**

Values are presented as mean ± standard deviation. The p-value was determined using the Mann–Whitney *U* test, and the significance level was adjusted after applying the Bonferroni correction. Statistically significant differences were defined as p ≤ 0.025 when applying the Bonferroni correction for both DA 0.01 and OP, as well as for 30 Hz flicker in photopic ERG. For DA 3.0, 10.0, and LA 3.0, the level of significance was set at p < 0.012 Differences that reach statistical significance are presented in bold. *DM1* type 1 diabetes, *DA* dark adapted, *LA* light adapted.

The ICC value for the DM group in the mixed scotopic a and b waves was 0.328 and 0.512, respectively. For the control group the corresponding values were 0.416 and 0.481. The ICC values for both groups ranged between 0.41 and 0.6, indicating a moderate correlation. Both eyes were considered for the analyses.

A significant increase in the IT of the rod-initiated DA 0.01 b-wave were observed in type 1 diabetes patients compared to the control group (p = 0.017). The amplitude of the DA mixed response b-wave was statistically reduced in the DM1 group (p = 0.005), without differences in either the ITs or the a-wave amplitude. There was an increase in the b-wave IT in the mixed response at the higher stimulus intensity in the diabetic group compared to the control group (p = 0.004), but no changes were observed in the amplitude of the waves. There were also no differences in the mixed-response b/a ratio. OP2 showed a significant increase in the IT (p = 0.008) and a significant decrease in its amplitude (p = 0.002) in the diabetic group.

In the photopic ERG, the 3.0 b-wave amplitude was significantly reduced in the diabetic group (p < 0.001). There were no changes in their ITs. The 30 Hz flicker response showed a reduction in amplitude in the diabetic group (p = 0.021), with no differences in the IT.

In the correlation study, significant negative correlations were found between age and some of the wave amplitudes (b wave amplitude of DA 0.01, r =  − 0.345 p = 0.020; b wave of DA 10, r =  − 0.318 p = 0.033; OP2 amplitude, r =  − 0.334 p = 0.025 and flicker 3.0 amplitude r =  − 0.371 r = 0.012). Age was positively correlated with different ITs: DA 0.01 b wave IT, r = 0.458 p = 0.002; DA 3.0 a wave IT, r = 0.881 p =  < 0.001; DA 3.0 b wave IT, r = 0.308 p = 0.039; DA 10 a wave IT, r = 0.736 p < 0.0001; DA 10 b wave IT, r = 0.296 p = 0.048; OP2 IT, r = 0.618 p < 0.0001; LA 3.0 a wave IT, r = 0.545 p < 0.0001 and LA flicker IT, r = 0.366 p = 0.013).

HbA1c showed a significant positive correlation with the IT of the LA 3.0 b wave (r = 0.570, p = 0.002) and the flicker 30 Hz IT (r = 579, p = 0.002). It had a significant negative correlation with the flicker 30 Hz amplitude (r = − 0.492 p = 0.013).

No correlations were found between the ERG results and the time of the onset of the disease.

### RETeval portable ERG values (Table 3)

**Table 3:** ERG values obtained with the RETeval ERG following the ISCEV protocol and the DR assessment protocol in the diabetic and control groups.

		Control group n = 46	DM1 group n = 46	p Mann–Whitney *U* test	Bonferroni correction
		Mean ± SD	Mean ± SD		
Scotopic ERG					
DA 0.28 Td.s	b-wave (ms)	96.92 ± 12.85	98.02 ± 12.10	0.743	NS
	b-wave (µV)	63.36 ± 19.02	53.47 ± 18.06	0.009	**0.009**
	Pupil (mm)	5.03 ± 0.85	4.84 ± 1.02	0.233	NS
DA 85 Td.s	a-wave (ms)	15.76 ± 1.90	16.12 ± 1.96	0.368	NS
	a-wave (μV)	− 51.21 ± 12.70	− 49.14 ± 13.61	0.324	NS
	b-wave (ms)	49.92 ± 5.12	49.86 ± 7.10	0.759	NS
	b-wave (µV)	96.53 ± 26.31	87.44 ± 23.34	0.181	NS
	Pupil (mm)	5.03 ± 0.83	4.68 ± 1.13	0.099	NS
DA 280 Td.s	a-wave (ms)	11.97 ± 0.95	12.54 ± 1.00	0.005	**0.005**
	a-wave (μV)	− 65.39 ± 15.87	− 63.44 ± 17.31	0.403	NS
	b-wave (ms)	50.98 ± 5.14	50.93 ± 7.52	0.773	NS
	b-wave (μV)	103.69 ± 25.89	92.44 ± 24.50	0.046	NS
	Pupil (mm)	5.05 ± 0.71	4.80 ± 0.97	0.208	NS
OP	OP2 (ms)	23.90 ± 1.78	24.97 ± 2.30	0.003	**0.003**
	OP2 (μV)	17.11 ± 7.10	12.45 ± 6.13	0.002	**0.002**
	Pupil (mm)	5.03 ± 0.83	4.68 ± 1.13	0.099	NS
Photopic ERG					
LA 85 Td.s	a-wave (ms)	11.76 ± 1.17	12.30 ± 2.88	0.506	NS
	a-wave (μV)	− 6.49 ± 2.64	− 6.44 ± 2.51	0.921	NS
	b-wave (ms)	29.74 ± 1.05	29.46 ± 1.14	0.252	NS
	b-wave (µV)	30.37 ± 13.22	26.89 ± 8.11	0.536	NS
	Pupil (mm)	2.13 ± 0.37	2.50 ± 0.73	0.010	**0.010**
28.3 Hz flicker	N1P1 (ms)	26.08 ± 1.30	26.03 ± 1.04	0.933	NS
	N1P1 (μV)	30.26 ± 10.75	27.54 ± 8.32	0.442	NS
	Pupil (mm)	1.94 ± 0.29	2.21 ± 0.62	0.002	**0.002**

Values are presented as mean ± standard deviation. The p-value was determined using the Mann–Whitney *U* test, and the significance level was adjusted after applying the Bonferroni correction. Statistically significant differences were considered with p ≤ 0.025 when applying the Bonferroni correction for both DA 0.28 Td.s OP and 28.3 Hz flicker. For DA 85 Td.s, 280 Td.s, LA 85 Td.s, 10.0, and LA 3.0, the level of significance was set at p < 0.012. Differences that reach statistical significance are presented in bold. *DM1* type 1 diabetes, *DA* dark adapted, *LA* light adapted.

In the DA 0.28 Td.s, the diabetic patients showed a diminished b-wave amplitude compared to the control group (53.47 ± 18.06 µV vs. 63.36 ± 19.02 µV, p = 0.009). The IT was similar in both groups. We did not find differences between the groups in the mixed-response DA. However, under the higher stimulus intensity (DA 280 Td.s), there was an increase in the a-wave IT (12.54 ± 1.00 ms vs. 11.97 ± 0.95 ms, p = 0.005) of the diabetic group compared to the control group, with no differences in either the b-wave IT or the b-wave and a-wave amplitude.

The diabetic patients demonstrated a significant decrease in the sum of the OP amplitudes (OPsum) compared to the control group (44.87 ± 14.87 vs. 61.49 ± 14.45 µV, p < 0.001). There were no differences in the OPsum IT. In the individual OP analysis, in the DM1 group, OP2 had increased IT (p = 0.003) and decreased amplitude (p = 0.002). OP3 and OP4 were reduced in amplitude (p < 0.001 and p < 0.001), with no changes in IT. The OP1 and OP5 values were similar in both groups (Table 4).

**Table 4:** Oscillatory potentials 85 Td.s and 0.1 Hz values obtained with the RETeval ERG following the ISCEV protocol in the diabetic and control groups.

		Control group n = 46	DM1 group n = 46	p
		Mean ± SD	Mean ± SD	
OP 85 Td∙s at 0.1 Hz				
OPsum	OPsum (ms)	152.11 ± 13.63	154.90 ± 12.92	0.184
	OPsum (μV)	61.49 ± 14.45	44.87 ± 14.87	** < 0.001**
OP1	OP1 (ms)	17.02 ± 1.56	17.87 ± 1.71	0.022
	OP1 (μV)	14.79 ± 6.57	13.89 ± 5.3	0.479
OP2	OP2 (ms)	23.90 ± 1.78	24.97 ± 2.3	**0.003**
	OP2 (μV)	17.11 ± 7.10	12.45 ± 6.13	**0.002**
OP3	OP3 (ms)	30.96 ± 2.38	31.78 ± 2.73	0.037
	OP3 (μV)	13.56 ± 4.98	8.51 ± 4.70	** < 0.001**
OP4	OP4 (ms)	38.35 ± 2.97	38.12 ± 5.56	0.476
	OP4 (μV)	8.89 ± 5.16	5.19 ± 3.12	** < 0.001**
OP5	OP5 (ms)	45.77 ± 3.48	45.32 ± 3.67	0.514
	OP5 (μV)	6.76 ± 4.68	5.75 ± 4.92	0.109
Pupil (mm)	Pupil (mm)	5.03 ± 0.83	4.68 ± 1.13	0.099

Values are presented as mean ± standard deviation. Differences were considered statistically significant with p < 0.005 when applying the Bonferroni correction. Differences that reach statistical significance are presented in bold. *DM1* type 1 diabetes, *OP* oscillatory potential, *OPsum* sum of oscillatory potentials.

For photopic ERG, both the LA 85 Td.s and the 28.3-Hz flicker showed no changes in the IT or in the amplitude. Pupil diameter was significantly longer in the type 1 diabetes group in the LA protocols (Table 3).

However, type 1 diabetes patients showed a significant increase in the IT and a significant decrease in the amplitude in the 28.3 Hz flicker at an illuminance of 16 Td.s (29.11 ± 1.69 vs. 28.42 ± 1.84 ms, p = 0.008 and 25.51 ± 8.01 vs. 21.58 ± 6.03 µV, p = 0.048, in the control and diabetic groups, respectively), with no differences at 32 Td.s illuminance. There were no changes in the pupil ratio of the DR assessment protocol, and the DR score was normal in both groups although the value was slightly higher in the diabetic group (18.05 vs. 17.39) (Table 5).

**Table 5:** ERG values obtained with the RETeval ERG for the DR assessment protocol in the diabetic and control groups.

		Control group n = 46	DM1 group n = 46	p
		Mean ± SD	Mean ± SD	
DR assessment				
16 Td.s 28.3 Hz	N1P1 (ms)	29.11 ± 1.69	28.42 ± 1.84	**0.008**
	N1P1 (μV)	25.51 ± 8.01	21.58 ± 6.03	**0.048**
32 Td.s 28.3 Hz	N1P1 (ms)	28.06 ± 1.52	27.71 ± 1.7	0.053
	N1P1 (μV)	29.97 ± 8.89	26.12 ± 7.59	0.088
Pupil area ratio	Diameter/mm	2.01 ± 0.43	2.00 ± 0.661	0.715
DR score	limits 7.0–23.4	17.39 ± 2.22	18.05 ± 2.47	0.411

Values are presented as mean ± standard deviation. Differences were considered statistically significant when p < 0.05. Differences that reach statistical significance are presented in bold. *DM1* type 1 diabetes, *DR* diabetic retinopathy.

The more marked correlations found with age were a significant negative correlation with b wave amplitude LA 85 Td.s (r = − 0.280, p = 0.016), 16 Td.s flicker (r = − 0.251, p = 0.029), and 32 Td.s flicker (r = − 0.888, p = 0.012) and a significant positive correlation with some of the ITs: the IT of DA a wave with a stimulus of 85 Td.s (r = 0.328, p = 0.004), with 280 Td.s (r = 0.429, p < 0.001), and of the OPsum (r = 0.358, p = 0.001), OP3 (r = 0.335, p = 0.003), and OP4 (r = 0.360, p = 0.001). The pupil ratio was also negatively correlated with age (r = − 0.378, p = 0.001).

The HbA1c level was positively correlated with the OP4 IT (r = 0.481, p = 0.017), the DA b wave IT of the 32 Td.s flicker (r = 0.497, p = 0.014), the LA b wave IT under a stimulus of 85 Td.s (r = 0.437, p = 0.037) and the b wave IT of the LA 28.5 Td.s flicker (r = 0.482, p = 0.020). HbA1c was negatively correlated with the OP5 amplitude (r = − 0.406, p = 0.049).

The years of disease evolution did not show any correlations.

### Comparison between both ffERG devices

**Figure 1:**
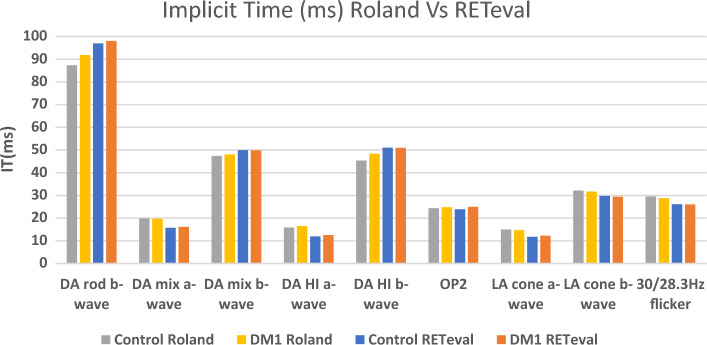
ERG implicit time values obtained with the Roland and RETeval recording systems in the control and diabetic groups. Values are expressed as means. *DM1* type 1 diabetes, *IT* implicit time, *DA* dark adapted, *HI* high intensity, *OP* oscillatory potentials, *LA* light adapted.

**Figure 2:**
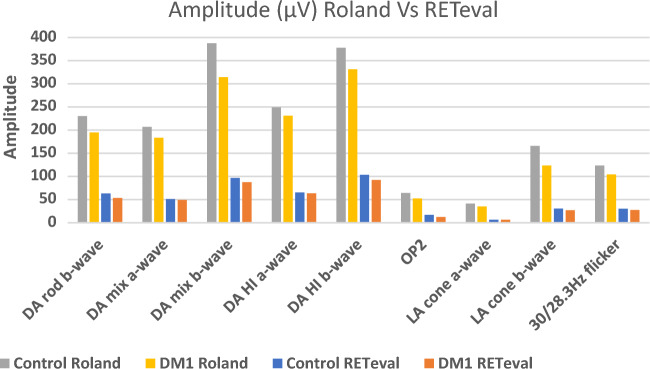
ERG amplitude values obtained with the Roland and RETeval recording systems for the control and diabetic groups. Values are expressed as means. *DM1* type 1 diabetes, *DA* dark adapted, *HI* high intensity, *OP* oscillatory potentials, *LA* light adapted.

**Figure 3:**
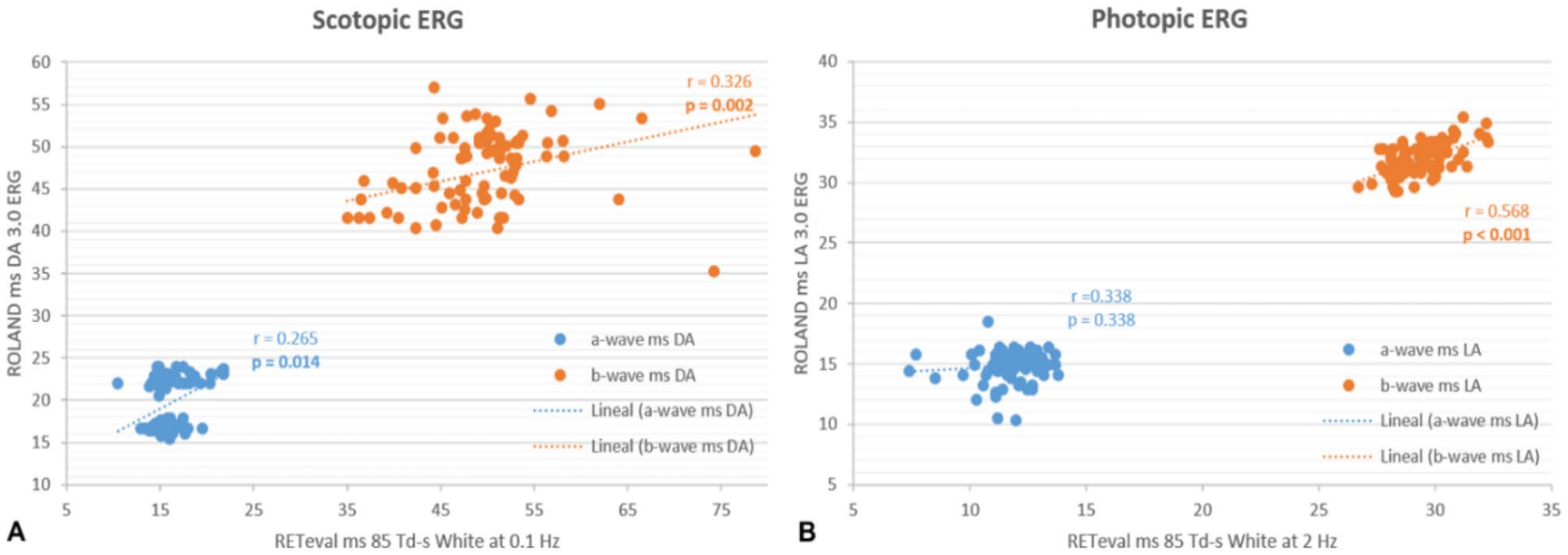
Scatter plots with regression lines, values of the correlation coefficients (r) and statistical significance (p) when comparing the corresponding values obtained with the two ERGs. (**A**) In mixed-wave dark-adapted (DA) conditions, correlation of the implicit time (IT) in ms of the a-wave (blue) and b-wave (orange) obtained with the RETeval vs. Roland. (**B**) In light-adapted (LA) conditions, correlation of the IT in ms of the a-wave (blue) and b-wave (orange) obtained with the RETeval vs. Roland. Values of p < 0.05 are considered statistically significant and are marked in bold.

**Figure 4:**
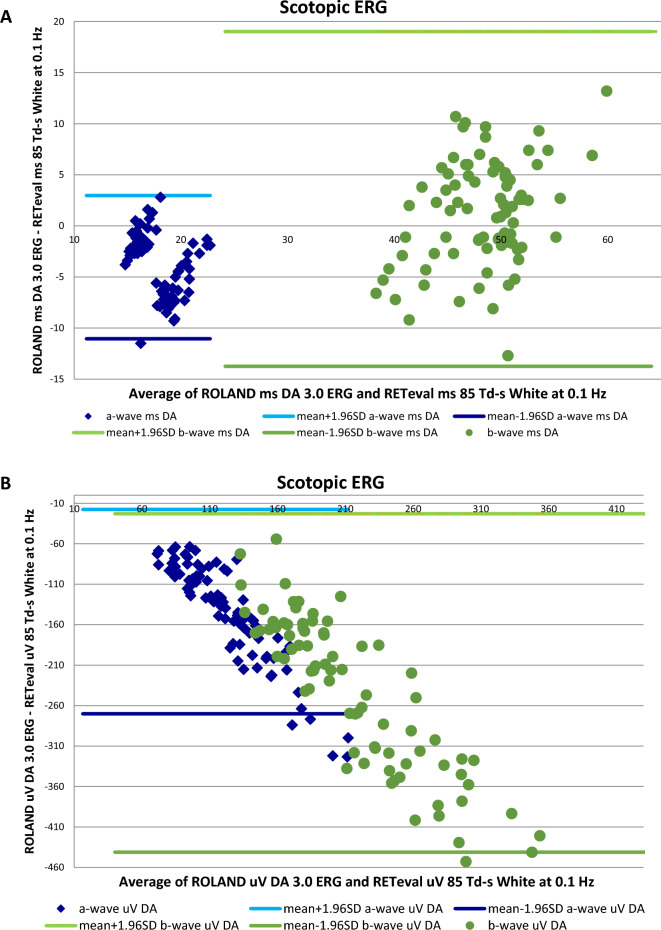
Bland–Altman plots for the a and b-wave IT obtained in DA mixed response and photopic stimulus for both ERG devices. (**A**) Mixed-wave dark-adapted (DA) conditions, correlation of the implicit time (IT) in ms of the a-wave (blue) and b-wave (orange) obtained with the RETeval vs. Roland. (**B**) In light-adapted (LA) conditions, correlation of the IT in ms of the a-wave (blue) and b-wave (orange) obtained with the RETeval vs. Roland.

**Figure 5:**
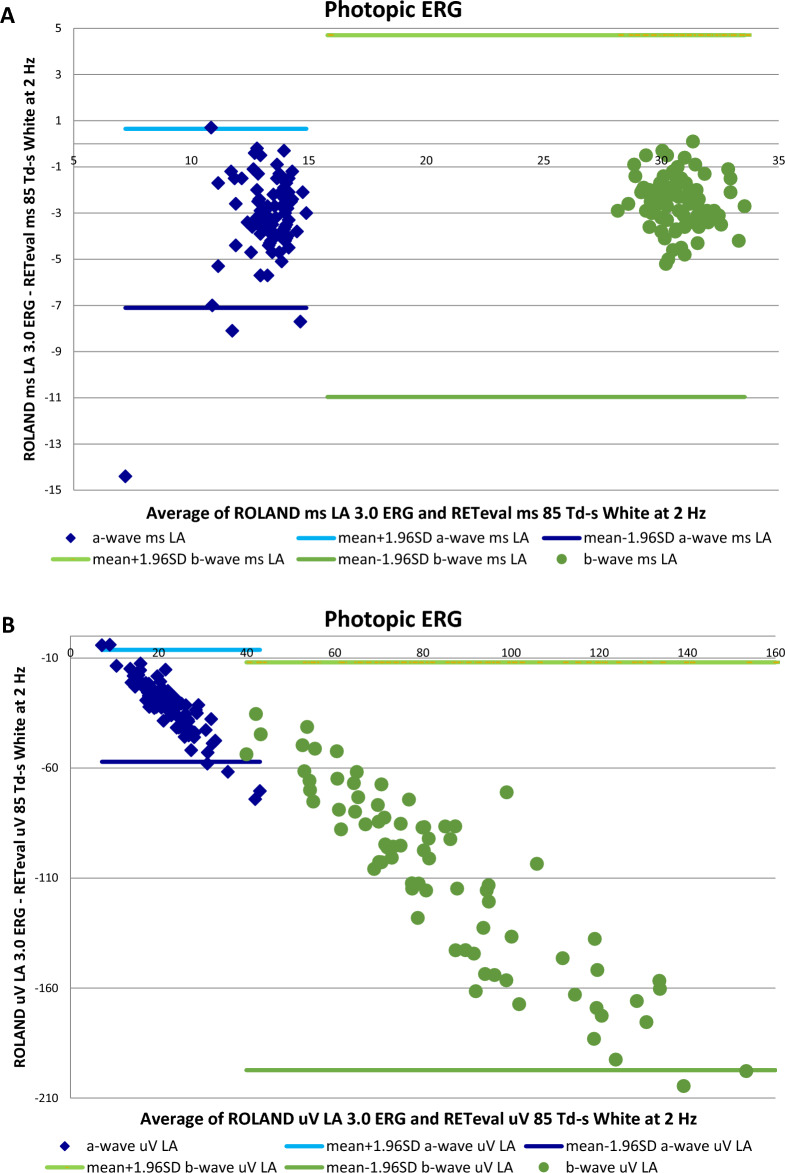
Bland–Altman plots for the a and b-wave amplitude obtained in DA mixed response and photopic stimulus for both ERG devices. (**A**) Mixed-wave dark-adapted (DA) conditions, correlation of the implicit time (IT) in ms of the a-wave (blue) and b-wave (orange) obtained with the RETeval vs. Roland. (**B**) In light-adapted (LA) conditions, correlation of the IT in ms of the a-wave (blue) and b-wave (orange) obtained with the RETeval vs. Roland.

There were differences in the values acquired by both ERG devices (Figs. 1, 2), but significant positive correlations were found between almost all the corresponding stimuli and responses when using both ffERG recording systems in the whole study sample (n = 88) (Table 6, Figs. 3, 4, 5). Scatter plots and Bland–Altman plots were employed to assess agreement in both latency and amplitude of the a and b waves in the mixed scotopic response and LA response, using 3.0 and 85 Td.s stimuli with both RETI-port and RETeval devices. Figure 3 displays the scatter plot with regression lines for both devices and Figs. 4 and 5 illustrate the agreement between the two devices in scotopic and photopic conditions, as well as in both IT and amplitude of the a and b waves. We could see that almost all the measurements of both IT and amplitude of both waves were between the mean ± 1.96 SD.

**Table 6:** Significant differences (p values) between the diabetic group and the control group obtained with both ffERG systems (left chart).

n = 88		ROLAND control vs. DM1 groups	RETeval control vs. DM1 groups	Correlation coefficient (r) between devices	p
Scotopic ERG					
DA rod response	b-wave (IT)	**0.017**		0.254	**0.018**
	b-wave (Amp)	**0.036**	**0.009**	0.300	**0.006**
DA mixed response	a-wave (IT)			0.265	**0.014**
	a-wave (Amp)			0.180	0.098
	b-wave (IT)			0.326	**0.002**
	b-wave (Amp)	**0.005**		0.138	0.214
DA high intensity	a-wave (IT)	**0.023**	**0.005**	0.439	** < 0.001**
	a-wave (Amp)			0.224	**0.039**
	b-wave (IT)	**0.004**		0.423	** < 0.001**
	b-wave (Amp)		**0.046**	0.270	**0.012**
OP2	OP2 (IT)	**0.008**	**0.003**	0.383	** < 0.001**
	OP2 (Amp)	**0.002**	**0.002**	0.246	**0.023**
Photopic ERG					
LA cone response	a-wave (IT)			0.103	0.338
	a-wave (Amp)	**0.036**		0.019	0.862
	b-wave (IT)			0.568	** < 0.001**
	b-wave (Amp)	** < 0.001**		0.135	0.225
30/28.3 Hz flicker	N1P1 (IT)			0.730	** < 0.001**
	N1P1 (μV)	**0.021**		0.243	**0.028**

Correlation coefficients obtained between both ERG systems (right chart). Values that achieved statistical significance are expressed in bold. *DM1* type 1 diabetes, *DA* dark adapted, *IT* implicit time, *Amp* amplitude, *OP* oscillatory potentials, *LA* light adapted.

## Discussion

In the current study, we found functional changes in long-term type 1 diabetes patients without DR. Neurodegeneration causes functional and structural abnormalities prior to clinical signs of DR^7^. ERG dysfunction can be found before the onset of DR signs, as a marker of neuronal impairment. Recent ffERG findings have indicated the possibility that photoreceptor function is abnormal in early-stage disease^18^. In this study, we compared functional changes in long-standing diabetic patients without signs of DR using conventional ffERG according to the ISCEV protocol and using the RETeval portable recording system. Previous studies have shown that the RETeval can provide data that show retinal functional alterations in diabetes mellitus patients and that it is particularly useful as a screening tool^22,25,26,29–32^. The RETeval provides a retinal illuminating stimulation (in Td.s) by adjusting the brightness; this characteristic allows the device to obtain ffERG nonmydriatic recordings that are adjusted depending on pupillary diameter^33^.

We searched for differences in both systems, and although long-term type 1 diabetes patients without DR exhibited changes with both devices, their observations were not always the same. Differences in the IT obtained using RETeval can be correlated with the pupil diameter, as the RETeval IT is significantly correlated with the pupil size. Kato et al. found a positive correlation between the pupillary area and the IT^33^.

### Scotopic ERG

We observed a more affected ERG under DA. Given the high metabolic demand and elevated oxidative stress of the diabetic retina, particularly under DA conditions, it can be expected that DA ERG will be worse than photopic ERG^34^. In the DA rod response, we observed a significant increase in the IT of the b-wave in diabetic patients with the Roland system. However, when using the RETeval device, a significant decrease in the amplitude of the b wave was found, but no changes in its IT were observed. These results support previous evidence suggesting that the rod pathway response is affected in the early stages of diabetes mellitus. Luu et al. found an impaired rod-derived ERG both in amplitude and IT with preserved cone ERG in type 2 diabetes patients with no DR or NPDR compared to controls^35^.

Previous studies show little or no changes in the a and b waves of the DA mixed response in patients without DR following ISCEV recommendations^36–38^. Lecleire-Collet et al. showed a reduction in the amplitude of the mixed scotopic b wave, both a and b wave photopic single flash, OPs and an altered vascular response to flicker in 28 DM patients without DR^39^. We also observed a decrease in the b wave amplitude of the combined response using the Roland system. With the RETeval system, diabetic patients showed a lower amplitude of the b wave without achieving statistical significance.

A statistically significant delay of the b-wave was recorded using the Roland system after the higher DA stimulus. Using the RETeval system, the a-wave was delayed. In addition, both systems showed a decrease in the amplitude of the b wave, although this did not reach statistical significance.

OPs can disappear in diabetes mellitus patients without DR and have been found to be an important tool in predicting the rate of progression of the disease^40^. We found OP changes in the type 1 diabetes group with both ffERG systems. They had decreased amplitudes and prolonged IT compared to controls. These results are consistent with previous studies suggesting that OPs are the ERG parameters that are constantly altered before vasculopathy appears; a delayed IT has been related to RD development or progression^18,35,39,41,42^. This observation could be related to hypoxia since amacrine cell-mediated OPs are highly sensitive to changes in retinal vascularization^16^. OPs are thought to reflect activity within the inner retina and are related to interactions between bipolar, amacrine and ganglion cells in the inner retina^43^. The exact cellular contribution to each individual OP is not clear, and the OP responses are affected differently in a diseased retina^35,43,44^.

Furthermore, the results of the individual analysis using RETeval of the OPs are consistent with previous studies suggesting that the first ones to disappear are OP2 and OP3, whereas OP4 tends to vanish in more extensive injuries^44^.

### Photopic results

Numerous studies have evaluated the photopic response in diabetic patients without DR and have found no changes in early stages with the traditional stimuli recommended by the ISCEV; light-adapted abnormalities are more evident in patients with advanced DR^18,36,45,46^. In our study, we observed a statistically significant decrease in photopic b-wave amplitude with the Roland system. In contrast, the RETeval system did not register significant changes in these conditions. Kizawa et al. found no photopic changes but described a decrease in both OPs and PhNR with the progression of DR^36^. Chen et al. also described the PhNR as a sensitive indicator of inner retinal function in diabetes mellitus patients^45^. Cone sensitivity reduction could be checked using high-frequency flicker or stimuli with different colours. McAnany et al. demonstrated these changes with a 62.5 Hz flicker^47^. Yamamoto et al. demonstrated a selective reduction in S cone ERG with a normal M-L cone response using chromatic stimuli in both patients with and without DR (15 out of 31) compared to normal subjects^48^ due to the higher susceptibility of S cones to hypoxic damage.

### Flicker

Using a 30 Hz flicker, as suggested by ISCEV, the response is produced by both on (depolarizing) and off (hyperpolarizing bipolar cells) pathways. With Ganzfeld stimulation, a 30 Hz flicker is typically found to be normal or only minimally reduced in individuals who have mild or no DR or an increase in its peak latency^18,47^. Neurovascular coupling has been shown to deteriorate in the early stages of diabetes mellitus. Flicker-induced retinal vessel changes can be recognized by modifications in vascular dilation after flicker stimulation. Garhöfer demonstrated a diminished response of the retinal arteries to flicker stimulation in diabetes mellitus patients with no or mild NPDR using a retinal vessel analyser and 8 Hz flicker^49^. Other authors have also shown a decrease in flicker-induced retinal vascularization in patients without DR, which deteriorates as the disease appears^39,50^. In our study, we found a statistically significant decrease in amplitudes in the ISCEV flicker with the Roland system. Higher-frequency flicker has been shown to be diminished^47^ secondary to alterations in quantum absorption and/or retinal hypoxia. These abnormalities in high-frequency flicker would be related to an abnormal off bipolar cell function^51^. Jansson et al. showed a decreased amplitude and an IT increase in the photopic ERG study cohort of 151 DM1 patients. This association disappeared after adjusting for age and excluding patients who had undergone laser treatment. However, the mean retinal thickness was associated with the b-wave amplitude of the photopic cone response and the 30 Hz flicker, showing no correlation with retinopathy severity^46^.

### DR assessment protocol

We found an increase in IT and a decrease in amplitude after 16 Td.s stimulation. No changes were observed after 32 Td.s between groups. The RETeval device combines several measurements to generate a score that can be abnormal in diabetic patients who do not have DR, and the score increases as the DR severity progresses^18^. McAnany et al. found a 12% reduction in amplitude and a 5% delay in IT in diabetics compared to controls after pooling all the studies of diabetic patients with no DR signs or mild lesions^18,30,37,47,52^. We did not find differences in the DR score between the groups in our study, although the value was slightly higher in the diabetic group (18.05 vs. 17.39). Zeng et al. investigated the correlation among inner retinal thickness measured by OCT, vessel density evaluated by OCT angiography, and functional impairment with the RETeval using both 16 and 32 Td.s flicker. They observed a delayed IT in both DA flickers, which correlated with higher levels of HbA1c. However, our study did not replicate these findings, as we only observed changes in both IT and amplitude with the 16 Td.s stimulation. Additionally, Zeng et al. reported differences in both DR score and pupil area compared to the control group. Importantly, the delay in IT observed by them was not correlated with thickness changes in the ganglion cell layer^52^.

The RETeval DR risk assessment protocol has proven to be a good tool for DR screening, with an increase in the score with the progression of DR. However, it can be less useful at detecting early stages of the disease^53^.

The pupil area ratio is evaluated as part of the DR score. There were no differences in the pupil area ratio between the groups. In our study, type 1 diabetes patients showed a smaller pupil diameter in scotopic conditions, though this was not statistically significant, but higher diameters in photopic conditions, which was statistically significant, both in LA 85Td.s and 28.3 Hz flicker (p = 0.01 and 0.002, respectively). Patients with DR tend to have pupils with less variability in size than healthy subjects.

Our study did not intend to evaluate diagnostic accuracy in a clinical context. Nevertheless, we did compare the performance of the two ERG devices in terms of agreement and correlation, offering valuable insights into their concordance within a non-disease population. The relationship between response size and observed agreement is illustrated in Figs. 3, 4 and 5.

A notable limitation of our study is the use of both eyes from each patient. The challenge lies in identifying DM1 patients with an extended disease duration and an absence of DR lesions. Eyes from the same patient are known to exhibit similar behaviours. The decision not to randomize one of the eyes was driven by our objective to augment the sample size. We used the intraclass correlation coefficient (ICC) to evaluate inter-eye correlation; as it was far from 1, we included both eyes for the analyses.

In conclusion, this study detected neurodegenerative alterations in patients with long-standing type 1 diabetes without DR, highlighting the importance of early evaluation of retinal function in these individuals. The results obtained using both evaluation systems revealed significant changes in the diabetic group compared to controls, underscoring the utility of these tools in assessing retinal function.

Scotopic responses exhibited more anomalies than photopic responses, the OPs being particularly sensitive at detecting alterations in early stages of type 1 diabetes, even in the absence of DR, supporting earlier findings. There are differences between systems tested, but they exhibit a good correlation between almost all the studied parameters.

## Data Availability

The datasets generated and analysed during the current study are not publicly available to protect study participant privacy but are available from the corresponding author on reasonable request.

## References

[1] Klein BE (2007). Overview of epidemiologic studies of diabetic retinopathy. Ophthalmic Epidemiol..

[2] Schmier JK, Covert DW, Lau EC, Matthews GP (2009). Medicare expenditures associated with diabetes and diabetic retinopathy. Retina.

[3] Simó R, Stitt AW, Gardner TW (2018). Neurodegeneration in diabetic retinopathy: Does it really matter?. Diabetologia.

[4] Solomon SD (2017). Diabetic retinopathy: A position statement by the american diabetes association. Diabetes Care.

[5] Newman EA (2013). Functional hyperemia and mechanisms of neurovascular coupling in the retinal vasculature. J. Cereb. Blood Flow Metab..

[6] van Dijk HW (2009). Selective loss of inner retinal layer thickness in type 1 diabetic patients with minimal diabetic retinopathy. Investig. Opthalmol. Vis. Sci..

[7] Pinilla I (2020). Changes in total and inner retinal thicknesses in type 1 diabetes with no retinopathy after 8 years of follow-up. Retina.

[8] Sohn EH (2016). Retinal neurodegeneration may precede microvascular changes characteristic of diabetic retinopathy in diabetes mellitus. Proc. Natl. Acad. Sci..

[9] Barber AJ (1998). Neural apoptosis in the retina during experimental and human diabetes: Early onset and effect of insulin. J. Clin. Invest..

[10] Ewing FME, Deary IJ, Strachan MWJ, Frier BM (1998). Seeing beyond retinopathy in diabetes: Electrophysiological and psychophysical abnormalities and alterations in vision. Endocr. Rev..

[11] Sokol S (1985). Contrast sensitivity in diabetics with and without background retinopathy. Arch. Ophthalmol..

[12] Henson DB, North RV (1979). Dark adaptation in diabetes mellitus. Br. J. Ophthalmol..

[13] Pinilla I (2013). Changes in frequency-doubling perimetry in patients with type I diabetes prior to retinopathy. Biomed. Res. Int..

[14] Montesano G (2017). Structure–function relationship in early diabetic retinopathy: A spatial correlation analysis with OCT and microperimetry. Eye.

[15] Orduna-Hospital E (2021). Microperimetry and optical coherence tomography changes in type-1 diabetes mellitus without retinopathy. Diagnostics.

[16] Tzekov R, Arden G (1999). The electroretinogram in diabetic retinopathy. Surv. Ophthalmol..

[17] Bearse MA (2006). A multifocal electroretinogram model predicting the development of diabetic retinopathy. Prog. Retin. Eye Res..

[18] McAnany JJ, Persidina OS, Park JC (2022). Clinical electroretinography in diabetic retinopathy: A review. Surv. Ophthalmol..

[19] Bearse MA, Han Y, Schneck ME, Adams AJ (2004). Retinal function in normal and diabetic eyes mapped with the slow flash multifocal electroretinogram. Investig. Opthalmol. Vis. Sci..

[20] Pescosolido N, Barbato A, Stefanucci A, Buomprisco G (2015). Role of electrophysiology in the early diagnosis and follow-up of diabetic retinopathy. J. Diabetes Res..

[21] Maa AY (2016). A novel device for accurate and efficient testing for vision-threatening diabetic retinopathy. J. Diabetes Complicat..

[22] Değirmenci MFK, Demirel S, Batıoğlu F, Özmert E (2018). Role of a mydriasis-free, full-field flicker ERG device in the detection of diabetic retinopathy. Doc. Ophthalmol..

[23] Robson AG (2022). ISCEV standard for full-field clinical electroretinography (2022 update). Doc. Ophthalmol..

[24] McCulloch DL (2015). ISCEV standard for full-field clinical electroretinography (2015 update). Doc. Ophthalmol..

[25] Zeng Y (2020). Screening for diabetic retinopathy in diabetic patients with a mydriasis-free, full-field flicker electroretinogram recording device. Doc. Ophthalmol..

[26] Fukuo M (2016). Screening for diabetic retinopathy using new mydriasis-free, full-field flicker ERG recording device. Sci. Rep..

[27] Armstrong RA (2013). Statistical guidelines for the analysis of data obtained from one or both eyes. Ophthalmic Physiol. Opt..

[28] McAlinden C, Khadka J, Pesudovs K (2011). Statistical methods for conducting agreement (comparison of clinical tests) and precision (repeatability or reproducibility) studies in optometry and ophthalmology. Ophthalmic Physiol. Opt..

[29] Al-Otaibi H (2017). Validity, usefulness and cost of RET eval system for diabetic retinopathy screening. Transl. Vis. Sci. Technol..

[30] Zeng Y (2020). Retinal vasculature–function correlation in non-proliferative diabetic retinopathy. Doc. Ophthalmol..

[31] Brigell MG, Chiang B, Maa AY, Davis CQ (2020). Enhancing risk assessment in patients with diabetic retinopathy by combining measures of retinal function and structure. Transl. Vis. Sci. Technol..

[32] Zhu Y (2020). Different scan protocols affect the detection rates of diabetic retinopathy lesions by wide-field swept-source optical coherence tomography angiography. Am. J. Ophthalmol..

[33] Kato K, Kondo M, Sugimoto M, Ikesugi K, Matsubara H (2015). Effect of pupil size on flicker ERGs recorded with RET eval System: New mydriasis-free full-field ERG system. Investig. Opthalmol. Vis. Sci..

[34] Arden GB, Sivaprasad S (2012). The pathogenesis of early retinal changes of diabetic retinopathy. Doc. Ophthalmol..

[35] Luu CD, Szental JA, Lee S-Y, Lavanya R, Wong TY (2010). Correlation between retinal oscillatory potentials and retinal vascular caliber in type 2 diabetes. Investig. Opthalmol. Vis. Sci..

[36] Kizawa J, Machida S, Kobayashi T, Gotoh Y, Kurosaka D (2006). Changes of oscillatory potentials and photopic negative response in patients with early diabetic retinopathy. Jpn. J. Ophthalmol..

[37] Tyrberg M (2011). Electrophysiological studies in newly onset type 2 diabetes without visible vascular retinopathy. Doc. Ophthalmol..

[38] Longhin E (2016). Rod function in diabetic patients without and with early diabetic retinopathy. Eur. J. Ophthalmol..

[39] Lecleire-Collet A (2011). Evaluation of retinal function and flicker light-induced retinal vascular response in normotensive patients with diabetes without retinopathy. Investig. Opthalmol. Vis. Sci..

[40] Bresnick GH (1984). Electroretinographic oscillatory potentials predict progression of diabetic retinopathy. Arch. Ophthalmol..

[41] Li X, Sun X, Hu Y, Huang J, Zhang H (1992). Electroretinographic oscillatory potentials in diabetic retinopathy. Doc. Ophthalmol..

[42] Vadalà M, Anastasi M, Lodato G, Cillino S (2002). Electroretinographic oscillatory potentials in insulin-dependent diabetes patients: A long-term follow-up. Acta Ophthalmol. Scand..

[43] Wachtmeister L, Dowling JE (1978). The oscillatory potentials of the mudpuppy retina. Invest. Ophthalmol. Vis. Sci..

[44] Lachapelle P (1994). The human suprathreshold photopic oscillatory potentials: Method of analysis and clinical application. Doc. Ophthalmol..

[45] Chen H, Zhang M, Huang S, Wu D (2008). The photopic negative response of flash ERG in nonproliferative diabetic retinopathy. Doc. Ophthalmol..

[46] Jansson RW, Raeder MB, Krohn J (2015). Photopic full-field electroretinography and optical coherence tomography in type 1 diabetic retinopathy. Graefe’s Arch. Clin. Exp. Ophthalmol..

[47] McAnany JJ (2019). Amplitude loss of the high-frequency flicker electroretinogram in early diabetic retinopathy. Retina.

[48] Yamamoto S, Kamiyama M, Nitta K, Yamada T, Hayasaka S (1996). Selective reduction of the S cone electroretinogram in diabetes. Br. J. Ophthalmol..

[49] Garhofer G (2004). Reduced response of retinal vessel diameters to flicker stimulation in patients with diabetes. Br. J. Ophthalmol..

[50] Mandecka A (2007). Influence of flickering light on the retinal vessels in diabetic patients. Diabetes Care.

[51] Holopigian K, Greenstein VC, Seiple W, Hood DC, Carr RE (1997). Evidence for photoreceptor changes in patients with diabetic retinopathy. Invest. Ophthalmol. Vis. Sci..

[52] Zeng Y (2019). Early retinal neurovascular impairment in patients with diabetes without clinically detectable retinopathy. Br. J. Ophthalmol..

[53] Deng X (2021). A diagnostic model for screening diabetic retinopathy using the hand-held electroretinogram device RETeval. Front. Endocrinol..

